# Trends in cardiometabolic risk factors in the Americas between 1980 and 2014: a pooled analysis of population-based surveys

**DOI:** 10.1016/S2214-109X(19)30484-X

**Published:** 2019-12-12

**Authors:** NCD Risk Factor Collaboration (NCD-RisC)—Americas Working Group

## Abstract

**Background:**

Describing the prevalence and trends of cardiometabolic risk factors that are associated with non-communicable diseases (NCDs) is crucial for monitoring progress, planning prevention, and providing evidence to support policy efforts. We aimed to analyse the transition in body-mass index (BMI), obesity, blood pressure, raised blood pressure, and diabetes in the Americas, between 1980 and 2014.

**Methods:**

We did a pooled analysis of population-based studies with data on anthropometric measurements, biomarkers for diabetes, and blood pressure from adults aged 18 years or older. A Bayesian model was used to estimate trends in BMI, raised blood pressure (systolic blood pressure ≥140 mm Hg or diastolic blood pressure ≥90 mm Hg), and diabetes (fasting plasma glucose ≥7·0 mmol/L, history of diabetes, or diabetes treatment) from 1980 to 2014, in 37 countries and six subregions of the Americas.

**Findings:**

389 population-based surveys from the Americas were available. Comparing prevalence estimates from 2014 with those of 1980, in the non-English speaking Caribbean subregion, the prevalence of obesity increased from 3·9% (95% CI 2·2–6·3) in 1980, to 18·6% (14·3–23·3) in 2014, in men; and from 12·2% (8·2–17·0) in 1980, to 30·5% (25·7–35·5) in 2014, in women. The English-speaking Caribbean subregion had the largest increase in the prevalence of diabetes, from 5·2% (2·1–10·4) in men and 6·4% (2·6–10·4) in women in 1980, to 11·1% (6·4–17·3) in men and 13·6% (8·2–21·0) in women in 2014). Conversely, the prevalence of raised blood pressure has decreased in all subregions; the largest decrease was found in North America from 27·6% (22·3–33·2) in men and 19·9% (15·8–24·4) in women in 1980, to 15·5% (11·1–20·9) in men and 10·7% (7·7–14·5) in women in 2014.

**Interpretation:**

Despite the generally high prevalence of cardiometabolic risk factors across the Americas, estimates also showed a high level of heterogeneity in the transition between countries. The increasing prevalence of obesity and diabetes observed over time requires appropriate measures to deal with these public health challenges. Our results support a diversification of health interventions across subregions and countries.

**Funding:**

Wellcome Trust.

## Introduction

With more than 41 million annual deaths, non-communicable diseases (NCDs) and their associated risk factors pose important challenges for global health.^1^, ^2^ In the Americas, almost 6 million of the 7 million deaths that occurred in 2017 were due to NCDs, 14% of the total global deaths due to NCDs.^3^ Although countries in some subregions of the Americas share many cultural, economic, and developmental similarities, differences in urbanisation, pace, and timing of the demographic, nutritional, and epidemiological transitions have led to high heterogeneity in the burden of NCDs across countries in the region. In 2017, the age-standardised mortality rate for NCDs in the region ranged from less than 350 deaths per 100 000 people in Peru, Colombia, and Canada to more than 890 deaths per 100 000 people in Haiti, more than that observed in any other country in the region in 1990.^2^

As part of the challenges posed by the increasing burden of NCDs in the region, the plan of action of the Pan American Health Organization strategy for the prevention and control of NCDs, 2021–25, was set with the aim to reduce avoidable mortality and morbidity associated with NCDs, and to minimise the exposure to risk factors, through a series of actions at the regional and national levels. This initiative aligned with the WHO NCD Global Monitoring Framework and Global Action Plan 2013–20.^4^, ^5^

Describing the trends of cardiometabolic risk factors associated with NCDs is crucial for monitoring progress, planning prevention, and providing evidence to support policy efforts.^5^, ^6^ Previous analyses have either focused on the trends and burden of single risk factors in the region,^7^, ^8^, ^9^, ^10^ or on cross-sectional analyses without exploring changes over time.^11^ Given the relevance of these risk factors for health policy and decision-making, and focusing exclusively on the Americas, we aimed to analyse national and subregional trends, from 1980 to 2014, in mean body-mass index (BMI), obesity, mean systolic blood pressure, raised blood pressure, and diabetes in adults.

**Research in context**

**Evidence before this study**

We searched MEDLINE (using PubMed) using the terms ((hypertension[Title] OR “blood pressure”[Title]) OR (diabetes[Title] OR “type 2 diabetes”[Title]) OR (BMI[Title] OR “body mass index”[Title])) AND (“Americas”[Mesh]). We did not find national estimates for all risk factors reported in this study (ie, studies were largely done in limited regions or sites). The search yielded two reviews of diabetes and obesity prevalence in Latin American countries, published in 2002, indicating that an update of this evidence is warranted. Overall, the available evidence was limited to country-based research on the epidemiology of cardiometabolic risk factors. Comparable metrics between countries in the Americas region covering a large study period have not been published.

**Added value of this study**

This work provides the most comprehensive analysis of trends in cardiometabolic risk factors, namely body-mass index, blood pressure, and diabetes in the Americas. This research covers a substantially longer period (1980–2014) than that of any previous work and provides estimates for 37 countries and territories in the Americas. Our results suggest that the frequency of high body-mass index and diabetes has increased throughout the region and, conversely, the prevalence of raised blood pressure appears to have decreased over time. The findings suggest that countries with a high prevalence of a particular risk factor do not necessarily also have a high frequency of another risk factor. The results signal different patterns of convergence and divergence between risk factors within subregions.

**Implications of all the available evidence**

Despite the decline in the prevalence of raised blood pressure across the region, much work is still needed to curtail the increasing burden of high body-mass index and diabetes. Cardiometabolic risk factors are rising on the public health agenda of governments in the Americas, particularly among low-income and middle-income countries in the region. This momentum can benefit from the use of regional trends from this study, which will inform policy and action in the Americas.

## Methods

### Study design and data sources

Data used in this analysis were obtained from population-based surveys, pooled and analysed by the NCD Risk Factor Collaboration (NCD-RisC) and have been described in detail elsewhere (appendix p 2).^7^, ^8^, ^9^ Briefly, we accessed publicly available population-based multicountry and national measurement surveys (eg, Demographic and Health Surveys, Global School-based student Health Surveys, and surveys identified via the Inter-University Consortium for Political and Social Research and the European Health Interview and Health Examination Surveys Database) as well as the WHO STEPwise approach to Surveillance surveys. Anonymised individual record data from sources included in NCD-RisC were reanalysed by the Pooled Analysis and Writing Group or by data holders according to a common protocol.

We analysed national and regional trends in mean BMI, prevalence of obesity, mean systolic and diastolic blood pressure, prevalence of raised blood pressure (systolic blood pressure ≥140 mm Hg or diastolic blood pressure ≥90 mm Hg), and prevalence of diabetes (fasting plasma glucose ≥7·0 mmol/L, history of diabetes, or diabetes treatment) in adults aged 18 years and older, in the 37 countries of the Americas. For mean BMI and prevalence of obesity, results are reported for ages 20 years and older. Although the NCD-RisC has released estimates for BMI, prevalence of obesity, mean systolic and diastolic blood pressure, and prevalence of raised blood pressure up to 2016, estimates for diabetes are only available up to 2014. To maintain consistency in the analysis across the three risk factors, the study period was limited to 1980–2014. Additionally, although estimates for BMI prevalence were available for seven different categories (<18·5 kg/m^2^, 18·5 to <20 kg/m^2^, 20 to <25 kg/m^2^, 25 to <30 kg/m^2^, 30 to <35 kg/m^2^, 35 to <40 kg/m^2^, and ≥40 kg/m^2^), we report the prevalence of obesity (BMI ≥30 kg/m^2^) only, which is of key public health concern in the Americas.

Estimates were obtained using population-based data sources representative of national, subnational (ie, covering ≥1 subnational region, or >3 communities) or community (≤3 communities) levels, with measured height or weight, systolic or diastolic blood pressure, or at least one of the following diabetes biomarkers: fasting plasma glucose (FPG), oral glucose tolerance (OGTT), or HbA_1c_. Conversion of diabetes biomarkers to a common definition is described elsewhere.^9^

### Statistical analysis

Analyses were done separately for men and women, using a Bayesian model to obtain estimates by age group, country, and year. The model used here has been described and validated previously (appendix p 2).^7^, ^8^, ^9^, ^12^ The model had a hierarchical structure, in which estimates for each country and year were informed by their own data, data from other years in the same country, and data in other countries in the same region.^13^ The hierarchical structure shared information to a greater degree when data were non-existent or weakly informative (eg, a study with a small sample size is less informative than a study in the same country and year with a larger sample), and to a lesser extent for data-rich countries and subregions.

The model incorporated non-linear time trends and age patterns, allowing the age pattern to vary across countries. The model accounted for the possibility that the risk factors in subnational and community samples might systematically differ from nationally representative ones and have a larger variation than in national studies. These features were implemented by including data-driven fixed-effect and random-effect terms for subnational and community data. The fixed effects adjusted for systematic differences between subnational or community studies and national studies. The random effects allowed national data to have a larger influence on the estimates than those of subnational or community data with similar sample sizes. The model also accounted for rural–urban differences in risk factors, using data-driven fixed effects for rural-only and urban-only studies. These rural and urban effects were weighted by the difference between study-level and country-level urbanisation in the year when the study was done. The statistical model included country-level covariates that were specific to each of the risk factors: the proportion of the national population living in urban areas for BMI; average number of years of education, proportion of national population living in urban areas, a summary measure of the availability of different food types for human consumption for diabetes and blood pressure; and age-standardised adult mean BMI for diabetes.

We fitted this Bayesian model with the Markov chain Monte Carlo (MCMC) algorithm. We ran 55 000 iterations, monitoring convergence, and discarded 5000 to give 50 000 iterations. We then thinned the chains, to give 5000 iterations for each chain. The 20 chains were then combined, and further thinning was carried out to give the final 5000 samples from the posterior distribution of model parameters, which were then used to obtain the posterior distributions. The reported credible intervals (CIs) represent the 2·5th to 97·5th percentiles of the posterior distributions. Reported estimates were age-standardised using the WHO standard population.^14^ Results are presented at the subregional and country levels. Classification of countries into subregions (using posterior estimates) is described in the appendix (p 7) and classification of the countries is based on geographical, cultural, and epidemiological similarities among countries. To assess the correlation between risk factors, we used Spearman's correlation rank test for risk factor prevalence. Probabilities of increases or decreases in risk factors were calculated using the proportion of MCMC draws in which increases or decreases were observed.

### Role of the funding source

The funder of the study had no role in study design, data collection, analysis, interpretation, or writing of the report. The corresponding authors had full access to the data in the study. The corresponding authors had final responsibility for the decision to submit for publication.

## Results

**Figure 1:**
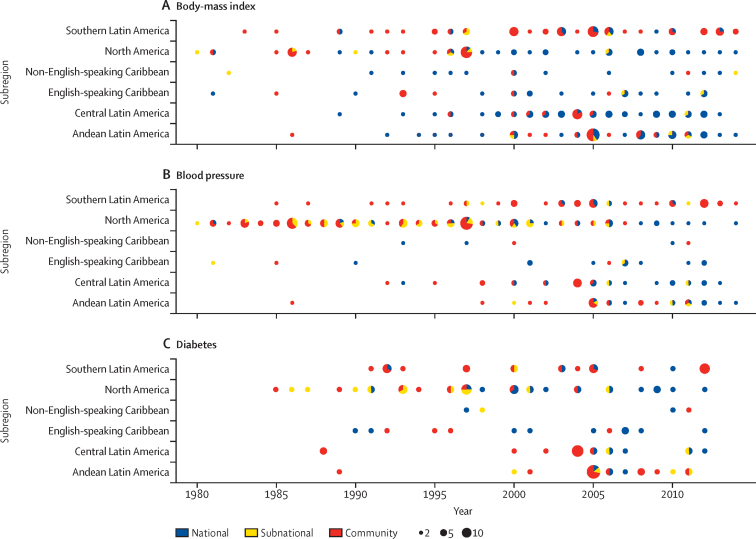
Number of data sources available for body-mass index, blood pressure, and diabetes by subregion and year

We used 389 population-based surveys from the Americas, of which 236 had information on BMI (1 783 267 participants), 223 had information on blood pressure (1 042 131 participants), and 108 had information on diabetes (284 555 participants). Among the 37 countries included, four (11%; Antigua and Barbuda, Bahamas, Bermuda, and Saint Vincent and the Grenadines) had no studies available (figure 1; appendix p 7).

**Figure 2:**
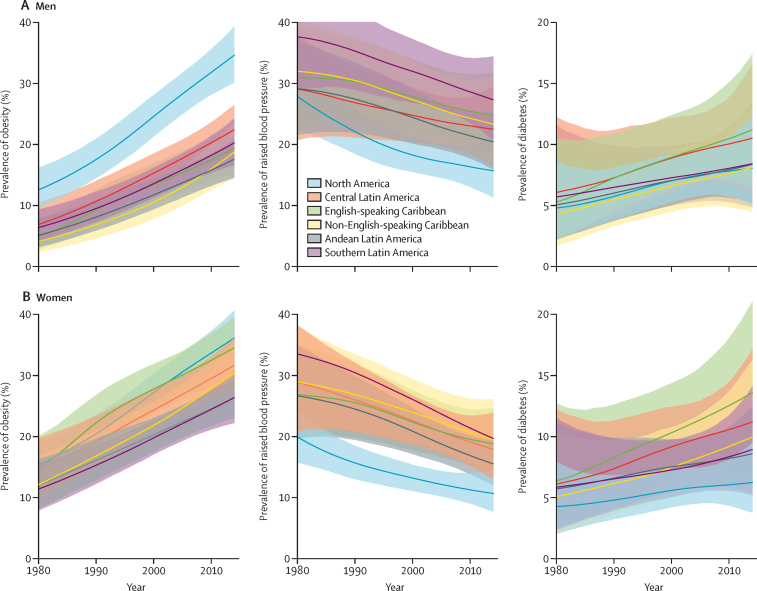
Trends in age-standardised prevalence of obesity, raised blood pressure, and diabetes by subregion in men and women The lines show the posterior mean estimates and the shaded areas show the 95% credible intervals.

From 1980 to 2014, the age-standardised mean BMI, and the age-standardised prevalence of obesity and diabetes increased in both sexes in all subregions in the Americas, whereas the age-standardised prevalence of raised blood pressure and the age-standardised mean systolic blood pressure declined in some subregions and did not change in others (figure 2; appendix pp 10, 11, 20, 21).

Obesity (and, similarly, mean BMI) increased everywhere in the region, with the fastest increase observed in the non-English-speaking Caribbean subregion, with the prevalence of obesity going from 3·9% (95% CI 2·2–6·3) in 1980, to 18·6% (14·3–23·3) in 2014, in men; and from 12·2% (8·2·17–0) in 1980, to 30·5% (25·7–35·5) in 2014, in women (figure 2). The English-speaking Caribbean subregion ranked first for prevalence of diabetes: 11·1% (6·4–17·3) in men and 13·6% (8·2–21·0) in women in 2014, with prevalence being 2·1 times larger (for men and women) in 2014 than in 1980 (figure 2). A decrease in raised blood pressure was observed in all subregions, regardless of the prevalence observed in 1980, with the largest decline being in North America, from 27·6% (22·3–33·2) in men and 19·9% (15·8–24·4) in women in 1980, to 15·5% (11·1–20·9) in men and 10·7% (7·7–14·5) in women in 2014 (figure 2; appendix pp 10–12).

Although mean systolic blood pressure has declined since the early 2000s, this trend appears to have stopped or reverted, particularly in North America, central Latin America, Andean Latin America, and southern Latin America for men and in North America and central Latin America for women (appendix pp 20, 21). Conversely, mean BMI increased almost linearly until the early 2000s, when this pace seems to have slowed down in North America for both men and women and in Central America and Andean Latin America for women (appendix pp 20, 21).

**Figure 3:**
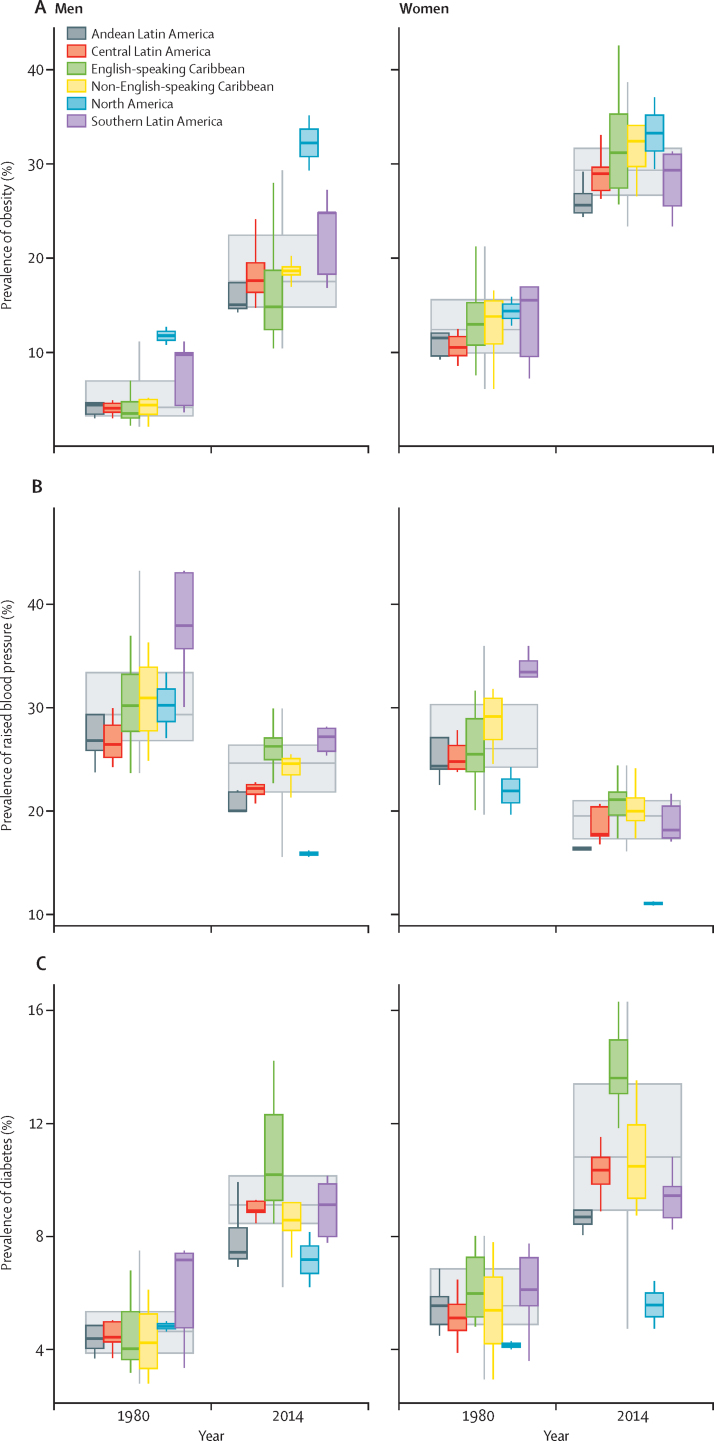
Boxplot showing the distribution of of country-level obesity, raised blood pressure, and diabetes prevalence Coloured boxes show how country-level prevalences are distributed within subregions (as opposed to population-weighted subregional means), and large uncoloured boxes show the country-level distributions for the Americas as a whole. Solid lines show medians, the boxes show IQRs, and the whiskers show ranges.

The analysis of risk factor trends over time was complemented with an assessment of convergence or divergence of disease burden by subregions. The consistent risk factor trends that were observed in the region hid non-uniform subregional trends (figure 3). We found a regional convergence for both sexes in the prevalence of raised blood pressure over time (figure 3B), with countries in North America showing a convergence towards much lower prevalence when compared with countries in the other subregions. A more complex pattern is observed in the prevalence of obesity (figure 3A) and diabetes (figure 3C), with a combination of subregional divergences, such as the variation in the prevalence of obesity in men or diabetes in women in all subregions.

**Figure 4:**
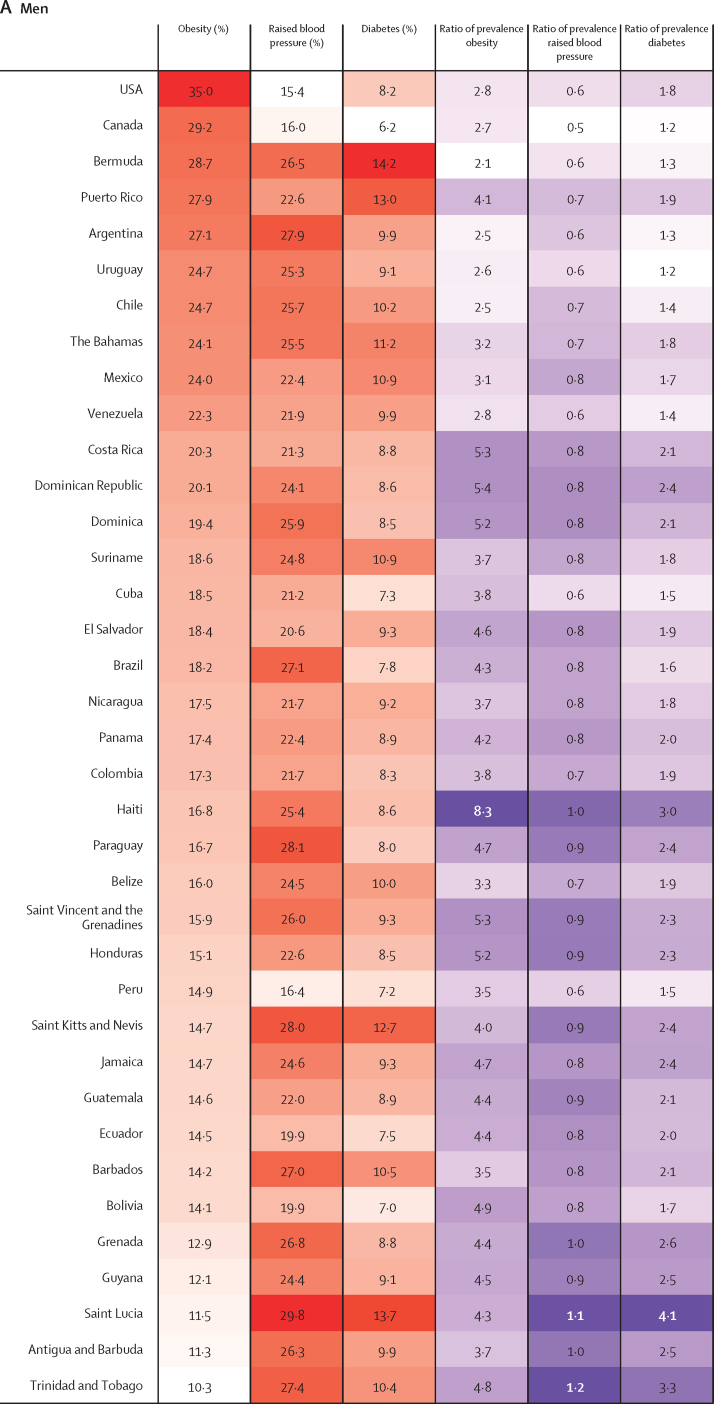

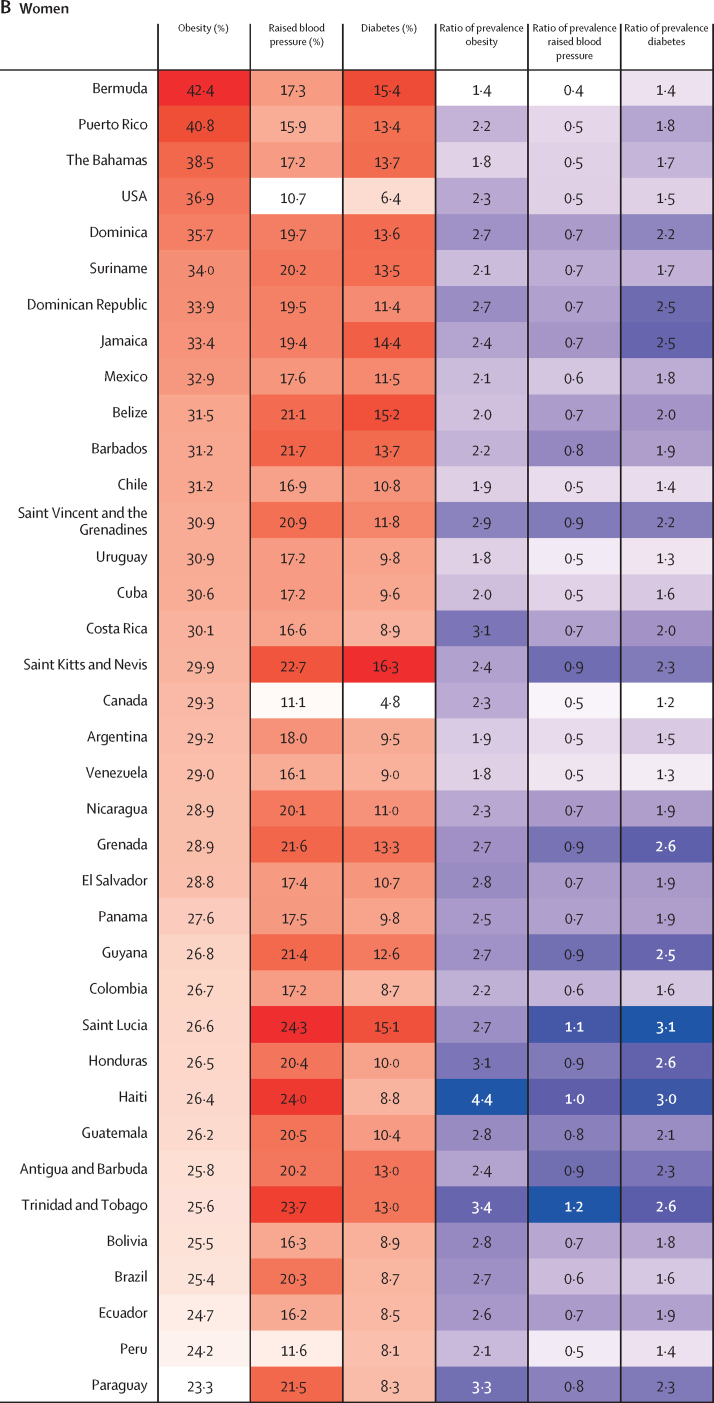
Heatmap of age-standardised prevalence of obesity, raised blood pressure, and diabetes by country in men and women in 2014, and proportional change from 1980 Countries are ranked by the prevalence of obesity. The ratio of prevalence for each risk factor are calculated for 2014 values relative to 1980 estimates. For the first three columns, red indicates the highest level in the prevalence of that specific risk factor and white the lowest; for the last three columns, purple indicates the highest ratio of prevalence and white the lowest.

The age-standardised prevalence of obesity, raised blood pressure, and diabetes for each country in 2014 are shown in figure 4 (appendix p 13), and corresponding estimates for 1980 are provided in the appendix (p 22). Country-specific trends are available online.^15^ From 1980 to 2014, the age-standardised prevalence of obesity in men increased in every country, with a range of 2·1–2·5 times in Bermuda, Argentina, and Chile (probability of an increase 0·999 in Bermuda, >0·999 in Argentina, and >0·999, in Chile), to 8·3 times in Haiti (>0·999; figure 4A). In women, the age-standardised prevalence of obesity increased by 1·4 to 1·8 times in Bermuda (0·99), the Bahamas (>0·999), Uruguay (>0·999), and Venezuela (0·999) and 4·4 times in Haiti (>0·999; figure 4B). However, for men in 2014, the prevalence of obesity was greater than 20% in almost a third of all countries, with the USA ranking first, with 35·0% (95% CI 30·1–40·1); whereas for women, all countries had a prevalence of obesity above 20%, with 16 (43%) of 37 having a prevalence above 30% (figure 4).

Changes in the prevalence of raised blood pressure between 1980 and 2014 included a 50% reduction in Canada in men (probability >0·999) and a 60% reduction in Bermuda (0·999) in women (figure 4). Most countries showed declines in the prevalence of raised blood pressure, and the only two countries where increases were noted were in Saint Lucia (10%, probability 0·75 for men and 0·63 for women) and in Trinidad and Tobago (20%, 0·72 for men and 0·74 for women) for both sexes (figure 4). In 2014, the countries within the English-speaking Caribbean and southern Latin America sub-regions (Saint Lucia, Paraguay, Saint Kitts and Nevis, and Argentina) had the highest prevalence of raised blood pressure in men (range 27·9–29·8%), whereas for women highest prevalence was in the countries in both Caribbean subregions (figure 4).

Relative to that of 1980, all countries showed an increase in the prevalence of diabetes in both sexes. Diabetes in men increased from 1·2 times in Canada (probability 0·75) and Uruguay (0·69) to 4·1 times in Saint Lucia (0·99; figure 4A), and diabetes in women increased from 1·2-times in Canada (0·70) to 3·1 times in Saint Lucia (0.98; figure 4B). Similar to those of other risk factors, the highest estimates for prevalence of diabetes, up to 14·2% for men and 16·3% for women, were concentrated in the English-speaking Caribbean countries in both sexes.

**Figure 5:**
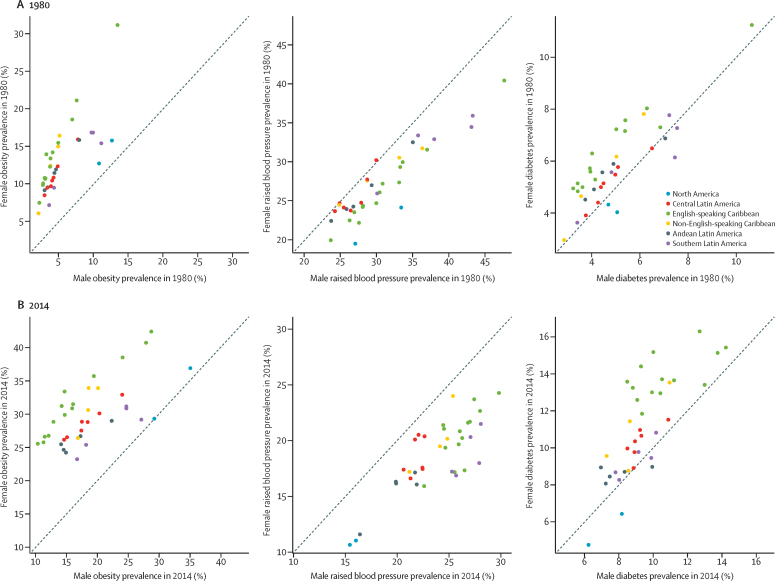
Male vs female age-standardised prevalence of obesity, raised blood pressure, and diabetes in 1980 and 2014

In 2014, women showed a higher prevalence of obesity and diabetes than men did, but a lower prevalence of raised blood pressure (figure 5B), except for in the USA, Canada, Argentina, and Venezuela, where the prevalence of raised blood pressure and diabetes was higher in men than in women (figure 5B). The difference between men and women in 2014 was larger than that in 1980 (figure 5A).

The comparison using Spearman's correlation rank test of risk factor prevalence revealed a positive correlation between obesity and raised blood pressure (*r*=0·75 for men and *r*=0·67 for women), between obesity and diabetes (*r*=0·88 for men and *r*=0·80 for women), and between diabetes and raised blood pressure (*r*=0·74 for both sexes) in 1980, but not in 2014 (*r*=-0·25 for obesity and raised blood pressure, *r*=0·053 for obesity and diabetes, and *r*=0·51 for diabetes and raised blood pressure in men, and *r*=–0·22 for obesity and raised blood pressure, *r*=0·51 for obesity and diabetes, and *r*=0·49 for obesity and raised blood pressure in women; appendix p 23).

## Discussion

This study provides a comprehensive assessment of obesity, raised blood pressure, and diabetes prevalence in the Americas between 1980 and 2014 and enhances our understanding of transitions in cardiometabolic risk factors in the region. Our estimates show that countries with the highest prevalence of a given cardiometabolic risk factor do not necessarily have the highest prevalence of another risk factor. The Americas has an obesity crisis accompanied by heterogeneous patterns of diabetes and raised blood pressure. These findings are important for the scoping and prioritisation of adequate NCD responses.

The increasing prevalence in obesity and diabetes observed in the region over time requires appropriate measures to deal with these public health challenges. The reasons behind the disparity in the associations between obesity and diabetes or between obesity and raised blood pressure between subregions and in the same subregion at different times are multiple. Traditionally, the term diabesity has denoted the epidemiological parallelism between obesity and diabetes, which is understandable considering the shared pathophysiological mechanisms.^16^ In terms of human capital, many countries in the Americas region are also transitioning from major burdens faced during childhood, particularly undernutrition.^17^, ^18^ With this transition, the long-lasting effects of early undernutrition, a direct cause of nutritional stunting, could play a role in developing NCDs later in life.^19^

Our study expands the regional literature because we have included all countries with data covering 35 years, a period that has witnessed important socioeconomic changes in the Latin American and Caribbean regions. Our analysis showed that the prevalence of cardiometabolic risk factors, with the exception of raised blood pressure, has steadily increased, and we predict that the prevalence to continue to increase. Whereas previous studies have mostly focused on South America,^20^, ^21^, ^22^, ^23^ we included all countries from Central America and the Caribbean, as well as the USA and Canada. The evidence provided highlights how the heterogeneity in the prevalence of risk factors requires multisectoral and locally adapted responses to improve prevention and treatment.

Given the high level of heterogeneity observed in the region, our results would support a diversification of health interventions across subregions and countries. For example, the focus in North America should be on reducing the level of obesity, whereas countries in the other subregions are experiencing a real emergency, with interventions needed not only to reduce the prevalence of obesity but also to prevent diabetes and raised blood pressure. Countries with a low prevalence of cardiometabolic risk factors, such as Peru, Bolivia, and Ecuador, should focus on how to reverse the increasing trends, continue with good profiles, and implement new policies.^24^ For example, compared with other countries in Africa (South Africa) and Asia (China, India, and Pakistan), some countries in South America (Argentina, Chile, Peru, and Uruguay) have better rates of awareness, treatment, and control of hypertension.^25^ However, inequalities within countries (and between countries) should be addressed to ensure that all population groups have these adequate patterns of awareness, treatment, and control of hypertension, such as in the USA, where Hispanic and black populations are characterised by different risk profiles when compared with the white population.^26^, ^27^ Such heterogeneity is further exemplified by the INTERHEART study, in which the population attributable risk for acute myocardial infarction due to diabetes ranged from 7·4 in Colombia to 17·0 in Brazil.^21^

Our estimates focused on raised blood pressure instead of hypertension, which in epidemiological studies is a composite variable, including measured blood pressure, a diagnosis of hypertension, or whether currently receiving treatment for hypertension. Our results should be interpreted in this context. Nevertheless, the analysis of trends in raised blood pressure is key for the WHO NCD Global Monitoring Framework because one of the nine voluntary global targets is “a 25% reduction in the prevalence of raised blood pressure”.^5^ The declining trends in the prevalence of raised blood pressure could signal improvements in the awareness, treatment, and control of hypertension, which seems to be the case in Canada^28^ and the USA,^27^ although we are unaware of similar trends for other countries and subregions in the region.

Our findings also show that the prevalence of the three cardiometabolic risk factors was highest in the small countries in the Caribbean subregion. Although countries in this subregion vary in economic terms, the absolute population size might contribute to these poor health outcomes.^29^, ^30^ With high prevalence and poor morbidity outcomes, this area is characterised by high levels of susceptibility, with limited ability to respond to the health needs of the population, including NCDs and chronic health issues, paired with limited health-care funding.

Tackling NCDs in the Americas will require high-risk approaches that are individual based, with population-based strategies.^31^, ^32^ This study provides sufficient data-driven insights for decision making and serves as a baseline to monitor and assess the effect of initiatives to be implemented in the region.^33^ Our analysis offers a comprehensive picture and improved visualisation of the particular features of the region in terms of major cardiometabolic risks. However, limitations should also be acknowledged. First, because of data availability, we did not include other important cardiometabolic risk factors such as waist circumference, smoking, or lipid profile. We presented results for established cardiometabolic risk factors, accounting for their availability in rural and resource-limited settings. Second, we did not include data from children or adolescents, and future efforts should include these populations because cardiometabolic risk factors have also probably increased among them.^34^, ^35^ Third, good quality nationally representative data on cardiometabolic risk factors are still absent in some countries, especially in the Caribbean. We recognise that estimates from pooled analyses might differ slightly from national surveys, especially in data-poor countries, and the intention of this exercise is not to supersede local efforts but to maximise available resources and to support local endeavours to produce current comprehensive data.

This region-wide analysis is the result of a collective approach, maximising the existing data available with a common goal for the public good. To overcome some of the limitations, and to support cardiometabolic prevention interventions that are effective, national and international support should be provided to improve the surveillance system, with cross-sectional and cohort studies aimed at monitoring cardiometabolic risk factors, especially in data-poor countries, which would benefit the most from improved estimates.

We addressed some of the most important risk factors for NCDs, closing the gap in the lack of information on the prevalence of cardiometabolic risk factors in the Americas. Despite the high prevalence of cardiometabolic risk factors in the Americas, estimates show substantial variation exists between countries and subregions. Countries with the highest prevalence of one risk factor do not necessarily also stand out for another risk factor, and men's profiles do not necessarily resemble those of women. Time trends show that, except for raised blood pressure, the prevalence of risk factors has steadily increased since 1980. Additionally, the within-region divergence in trends suggests that risk factors are not increasing or decreasing at the same pace in all countries. This study can signal opportunities for joint prevention efforts or interventions oriented to reduce common risk factors in the region, given the interconnectedness in cultural, social, demographic, ethnic, economic, and political aspects of the countries in the Americas.

Correspondence to: Prof J Jaime Miranda, CRONICAS Centre of Excellence in Chronic Diseases, and Department of Medicine, School of Medicine, Universidad Peruana Cayetano Heredia, Miraflores, Lima 18, Peru jaime.miranda@upch.pe

or

Dr Mariachiara Di Cesare, Department of Natural Sciences, Middlesex University, London NW4 4BT, UK c.dicesare@mdx.ac.uk

## References

[1] World Health Organization (2015). Noncommunicable Diseases Progress Monitor 2015. http://www.who.int/nmh/publications/ncd-progress-monitor-2015/en/.

[2] GBD Compare Institute for Health Metrics and Evaluation. https://vizhub.healthdata.org/gbd-compare/.

[3] Global Burden of Disease (GBD) Institute for Health Metrics and Evaluation. https://vizhub.healthdata.org/gbd-compare/.

[4] PAHO (2014). Plan of action for the prevention and control of noncommunicable diseases in the Americas 2013–2019. https://www.paho.org/hq/dmdocuments/2014/NCD-en-lowres.pdf.

[5] WHO (2013). Global action plan for the prevention and control of noncommunicable diseases 2013–2020.

[6] Legetic B, Medici A, Hernández-Avila M, Alleyne G, Hennis A (2016). Economic Dimensions of Non-Communicable Disease in Latin America and the Caribbean. Disease Control Priorities.

[7] NCD Risk Factor Collaboration (NCD-RisC) (2017). Worldwide trends in blood pressure from 1975 to 2015: a pooled analysis of 1479 population-based measurement studies with 19·1 million participants. Lancet.

[8] NCD Risk Factor Collaboration (NCD-RisC) (2016). Trends in adult body-mass index in 200 countries from 1975 to 2014: a pooled analysis of 1698 population-based measurement studies with 19·2 million participants. Lancet.

[9] NCD Risk Factor Collaboration (NCD-RisC) (2016). Worldwide trends in diabetes since 1980: a pooled analysis of 751 population-based studies with 4·4 million participants. Lancet.

[10] GBD 2015 Obesity Collaborators (2017). Health effects of overweight and obesity in 195 countries over 25 years. N Engl J Med.

[11] Miranda JJ, Herrera VM, Chirinos JA (2013). Major cardiovascular risk factors in Latin America: a comparison with the United States. The Latin American Consortium of Studies in Obesity (LASO). PLoS One.

[12] Finucane MM, Paciorek CJ, Danaei G, Ezzati M (2014). Bayesian estimation of population-level trends in measures of health status. Stat Sci.

[13] Stevens GA, Singh GM, Lu Y (2012). National, regional, and global trends in adult overweight and obesity prevalences. Popul Health Metr.

[14] Ahmad OB, Boschi-Pinto C, Lopez AD (2001). Age standardization of rates: a new WHO standard. GPE Discussion Paper Series: No 31 EIP/GPE/EBD.

[15] NCD-RisC Data visualisations. NCD Risk Factor Collaboration. http://ncdrisc.org/data-visualisations.html.

[16] Verma S, Hussain ME (2017). Obesity and diabetes: an update. Diabetes Metab Syndr.

[17] Corvalán C, Garmendia ML, Jones-Smith J (2017). Nutrition status of children in Latin America. Obes Rev.

[18] Smith LC, Haddad L (2015). Reducing child undernutrition: past drivers and priorities for the post-MDG era. World Dev.

[19] Sawaya AL, Martins PA, Grillo LP, Florêncio TT (2004). Long-term effects of early malnutrition on body weight regulation. Nutr Rev.

[20] Rubinstein AL, Irazola VE, Calandrelli M (2015). Multiple cardiometabolic risk factors in the Southern Cone of Latin America: a population-based study in Argentina, Chile, and Uruguay. Int J Cardiol.

[21] Lanas F, Avezum A, Bautista LE (2007). Risk factors for acute myocardial infarction in Latin America: the INTERHEART Latin American study. Circulation.

[22] Bautista LE, Casas JP, Herrera VM (2009). The Latin American Consortium of Studies in Obesity (LASO). Obes Rev.

[23] Schmidt MI, Duncan BB, Azevedo e Silva G (2011). Chronic non-communicable diseases in Brazil: burden and current challenges. Lancet.

[24] Salicrup L, Ordunez P, Engelgau M (2018). Hypertension control activities in Latin America and the Caribbean: opportunities for late-stage (T4) translation research. Rev Panam Salud Publica.

[25] Irazola VE, Gutierrez L, Bloomfield G (2016). Hypertension prevalence, awareness, treatment, and control in selected LMIC communities: results from the NHLBI/UHG network of centers of excellence for chronic diseases. Glob Heart.

[26] Fryar CD, Ostchega Y, Hales CM, Zhang G, Kruszon-Moran D (2017). Hypertension prevalence and control among adults: United States, 2015–2016. NCHS data brief, no 289.

[27] Egan BM, Zhao Y, Axon RN (2010). US trends in prevalence, awareness, treatment, and control of hypertension, 1988-2008. JAMA.

[28] Campbell NRC, Chen G (2010). Canadian efforts to prevent and control hypertension. Can J Cardiol.

[29] Economic Commission for Latin America and the Caribbean (ECLAC) (2018). The Caribbean outlook. https://repositorio.cepal.org/11362/43581.

[30] Hambleton IR, Howitt C, Jeyaseelan S (2015). Trends in longevity in the Americas: disparities in life expectancy in women and men, 1965–2010. PLoS One.

[31] GBD 2016 Mortality Collaborators (2017). Global, regional, and national under-5 mortality, adult mortality, age-specific mortality, and life expectancy, 1970–2016: a systematic analysis for the Global Burden of Disease Study 2016. Lancet.

[32] Mendoza W, Miranda JJ (2017). Global shifts in cardiovascular disease, the epidemiologic transition, and other contributing factors: toward a new practice of global health cardiology. Cardiol Clin.

[33] Hospedales CJ, Barcelo A, Luciani S, Legetic B, Ordunez P, Blanco A (2012). NCD prevention and control in Latin America and the Caribbean: a regional approach to policy and program development. Glob Heart.

[34] Rivera JÁ, de Cossío TG, Pedraza LS, Aburto TC, Sánchez TG, Martorell R (2014). Childhood and adolescent overweight and obesity in Latin America: a systematic review. Lancet Diabetes Endocrinol.

[35] NCD Risk Factor Collaboration (NCD-RisC) (2017). Worldwide trends in body-mass index, underweight, overweight, and obesity from 1975 to 2016: a pooled analysis of 2416 population-based measurement studies in 128·9 million children, adolescents, and adults. Lancet.

