# Correction to *Lancet Glob Health* 2020; 8: e123–33

**DOI:** 10.1016/S2214-109X(20)30107-8

**Published:** 2020-03-12

*NCD Risk Factor Collaboration (NCD-RisC)—Americas Working Group. Trends in cardiometabolic risk factors in the Americas between 1980 and 2014: a pooled analysis of population-based surveys.* Lancet Glob Health *2020; **8:** e123–33*. In this Article, members of the NCD Risk Factor Collaboration (NCD-RisC)—Americas Working Group have been indexed as authors in PubMed. This correction has been made as of March 12, 2020.

