# Correction to *Lancet Glob Health* 2020; 8: e123–33

**DOI:** 10.1016/S2214-109X(20)30516-7

**Published:** 2020-12-03

*NCD Risk Factor Collaboration (NCD-RisC)—Americas Working Group. Trends in cardiometabolic risk factors in the Americas between 1980 and 2014: a pooled analysis of population-based surveys.* Lancet Glob Health *2020; **8:** e123–33*—In this Article, working group member Luis Mascarenhas was incorrectly missing from the list of members and has been added to the list at the end of the manuscript. This correction has been made as of Dec 3, 2020.

