# Supplementary material for: Trends in cardiometabolic risk factors in the Americas between 1980 and 2014: a pooled analysis of population-based surveys
Source: Lancet Glob Health. 2019 Dec 12;8(1):e123–33. doi: 10.1016/S2214-109X(19)30484-X (PMC7025323; doi:10.1016/S2214-109X(19)30484-X)
Supplement: Supplementary appendix [file mmc1.pdf]

THE LANCET
Global Health

Supplementary appendix

This appendix formed part of the original submission and has been peer reviewed. We post it as supplied by the authors.

Supplement to: NCD Risk Factor Collaboration (NCD-RisC)—Americas Working Group. Trends in cardiometabolic risk factors in the Americas between 1980 and 2014: a pooled analysis of population-based surveys. *Lancet Glob Health* 2020; **8:** e123–33.

Transitions of cardio-metabolic risk factors in the Americas between 1980 and 2014

NCD Risk Factor Collaboration (NCD-RisC) – Americas Working Group

Appendix 1: Data sources, data management and statistical analysis 2

Table S1 – Number of data sources by country, sub-region and risk factor 7

Table S2 - Age-standardised prevalence of obesity (%), prevalence of raised blood pressure (%), and prevalence of diabetes (%) by sub-region in 1980 (95% CrI provided in brackets) 10

Table S3 - Age-standardised mean body mass index (BMI, kg/m2), prevalence of obesity (%), mean systolic blood pressure (mmHg), prevalence of raised blood pressure (%), and prevalence of diabetes (%) by sub-region in 2014 (95% CrI provided in brackets) 11

Table S4 – Prevalence of obesity (%), prevalence of raised blood pressure (%), and prevalence of diabetes (%), for men and women in 1980 and 2014, as well as the ratio of prevalence between 2014 and 1980 12

Table S5 - Age-standardised mean body mass index (BMI, kg/m2), prevalence of obesity (%), mean systolic blood pressure (mmHg), prevalence of raised blood pressure (%), and prevalence of diabetes (%) by country in 2014 (95% CrI provided in brackets) 13

Figure S1 – Mean body mass index and systolic blood pressure by sub-regions in men 20

Figure S2 – Mean body mass index and systolic blood pressure by sub-regions in women 21

Figure S3 – Heatmap of age-standardised prevalence of obesity, raised blood pressure (RBP), and diabetes (DM) by country in (A) men and (B) women in 1980. Countries ranked by prevalence of obesity 22

Figure S4 – Age standardised prevalence of obesity vs. age standardised prevalence of raised blood pressure and age standardised prevalence of diabetes by sex, (A) 1980 and (B) 2014 23

Appendix 1: Data sources, data management and statistical analysis

We used a database of population-based data on cardiometabolic risk factors collated by the NCD Risk Factor Collaboration (NCD-RisC), a worldwide network of health researchers and practitioners whose aim is to document systematically the worldwide trends and variations in non-communicable disease (NCD) risk factors. The database was collated through multiple routes for identifying and accessing data. We accessed publicly available population-based multi-country and national measurement surveys (e.g., Demographic and Health Surveys (DHS), Global School-based student Health Surveys (GSHS), and surveys identified via the Inter-University Consortium for Political and Social Research and the European Health Interview & Health Examination Surveys Database) as well as the World Health Organization (WHO) STEPwise approach to Surveillance (STEPS) surveys. We requested, via WHO and its regional and country offices, from ministries of health and other national health and statistical agencies to identify and access population-based surveys. Requests were also sent via the World Heart Federation to its national partners. We made a similar request to the co-authors of an earlier pooled analysis of cardiometabolic risk factors,[1-4] and invited them to reanalyse data from their studies and join NCD-RisC. Finally, to identify major sources not accessed through the above routes, we searched and reviewed published and invited all eligible studies to join NCD-RisC.[5-7]

Anonymised individual record data from sources included in NCD-RisC were reanalysed by the Pooled Analysis and Writing Group or by data holders according to a common protocol. Within each survey, we included participants aged five years and older who were not pregnant. We dropped participants with implausible values:

1. body mass index (BMI) levels (defined as BMI <7 kg/m^2 or BMI >80 kg/m^2 for children and adolescents and BMI <10 kg/m^2 or BMI >80 kg/m^2 for adults) or with implausible height or weight values (defined as height <80 cm, height >250 cm, weight <8 kg or weight >300 kg) (<0.2% of all subjects).
2. FPG (defined as FPG <2.5 mmol/L or FPG >30 mmol/L; <0.5% of all FPG data), 2hOGTT (2hOGTT <2 mmol/L or 2hOGTT >30 mmol/L; <0.2% of all 2hOGTT data), or HbA1c (HbA1c <3% or HbA1c >18%; <0.1% of all HbA1c data).
3. SBP (defined as SBP <70 mmHg or SBP >270 mmHg; 0.09% of all SBP data) or DBP (DBP <50 mmHg or DBP >150 mmHg; 0.07% of all DBP data).

We calculated:

1. Mean BMI, prevalence of a comprehensive set of BMI categories, and associated standard errors by sex and age group. For adults, we analysed prevalence in the following BMI ranges: <18·5 kg/m², 18·5 kg/m² to <20 kg/m², 20 kg/m² to <25 kg/m², 25 kg/m² to <30 kg/m², 30 kg/m² to <35 kg/m², 35 kg/m² to <40 kg/m², and ≥40 kg/m².
2. Mean SBP, mean DBP and prevalence of raised blood pressure defined as systolic blood pressure (SBP) ≥140 mmHg or diastolic blood pressure (DBP) ≥90 mmHg, and associated standard errors and sample sizes, by sex and age group (18-19 years, 20-29 years, followed by 10-year age groups and 80+ years). The NCD-RisC has made estimates for DBP itself; however, given that SBP and DBP are strongly correlated, we chose to report only SBP.
3. Prevalence of diabetes defined as FPG fasting plasma glucose ≥7.0 mmol/l, history of diabetes, or diabetes treatment in adults aged 18 years and older.

All analyses incorporated appropriate sample weights and complex survey design in calculating age-sex-specific means and prevalence, when applicable. To ensure summaries were prepared according to the study protocol, the Pooled Analysis and Writing Group provided computer code to NCD-RisC members who requested assistance. All submitted data were checked by at least two independent members of the Pooled Analysis and Writing Group. Questions and clarifications were discussed with NCD-RisC members and resolved before data were incorporated in the database.

Finally, we obtained data not accessed through the above routes by extracting from published reports of all additional national health surveys identified through the above-described strategies. Data were extracted from published reports only when reported by sex and in age groups no wider than 20 years. We also used data from a previous pooling study[1] when such data had not been accessed through the above routes.

NCD-RisC database is continuously updated through the above routes and through periodic requests to NCD-RisC members to help identify new data sources. In this paper, we used data from the NCD-RisC database for years 1975 to 2016. In this work we report estimates for adults aged 18+ years for blood pressure and diabetes, and adults aged 20+ for body mass index.

Data inclusion and exclusion

Data sources were included in NCD-RisC database if:

- measured data on height, weight; measured data on systolic blood pressure (SBP) and/or diastolic blood pressure (DBP); or measured data on fasting glucose, 2-h plasma glucose in an oral glucose tolerance test (2hOGTT) or haemoglobin A1c (HbA1c) were available;
- study participants were five years of age and older for anthropometric measurements and ten years of age and older for blood pressure and diabetes;
- data were collected using a probabilistic sampling method with a defined sampling frame;
- data were from population samples at the national, sub-national, or community level;
- data were collected in or after 1950.

We excluded all data sources that were solely based on self-reported measurements. We also excluded data sources on population subgroups whose specific measurements may differ systematically from the general population, including:

- studies that had included or excluded people based on their health status or cardiovascular risk;
- studies whose participants were only ethnic minorities;
- specific educational, occupational, or socioeconomic subgroups, with the exception noted below;
- those recruited through health facilities, with the exception noted below; and
- only for anthropometric measurements, women aged 15-19 years in surveys which sampled only ever-married women or measured height and weight only among mothers.

We used school-based data in countries, and in age-sex groups, with school enrolment of 70% or higher. We used data whose sampling frame was health insurance schemes in countries where at least 80% of the population were insured. Finally, we used data collected through general practice and primary care system in high-income countries with universal insurance, because contact with the primary care systems tends to be at least as good as response rates for population-based surveys.

Data management

For each data source accessed through the primaryand secondary data access process, we recorded

the available information about the study population, period of measurement, sampling approach, and

measurement methods. The information about study population was used to establish that each data source was population-based, and to assess whether it covered the whole country, multiple sub-national regions, or one or a small number of communities, and whether it was rural, urban, or combined.

We carefully checked all data sources in terms of how they met our inclusion and exclusion criteria. We identified duplicate data sources by comparing studies from the same country and year. Additionally, NCD-RisC members received the list of all data sources in the database and were asked to ensure that the included data from their country met the inclusion criteria and that there were no duplicates.

Statistical analysis

The model is fitted using a Markov chain Monte Carlo sampler coded in R, which uses a combination of Metropolis-Hastings and Gibbs updates. To ensure that the sampler has converged to the posterior distribution, we run 55,000 iterations and discard 5,000 to give 50,000 iterations. We then thin the chains, to give 5,000 iterations for each chain. The 20 chains are then combined, and further thinning is carried out to give the final 5,000 samples.

References

1. Finucane MM, Stevens GA, Cowan MJ, et al. National, regional, and global trends in body-mass index since 1980: systematic analysis of health examination surveys and epidemiological studies with 960 country-years and 9.1 million participants. *Lancet* 2011; **377**(9765): 557-67.

2. Danaei G, Finucane MM, Lin JK, et al. National, regional, and global trends in systolic blood pressure since 1980: systematic analysis of health examination surveys and epidemiological studies with 786 country-years and 5.4 million participants. *Lancet* 2011; **377**(9765): 568-77.

3. Danaei G, Finucane MM, Lu Y, et al. National, regional, and global trends in fasting plasma glucose and diabetes prevalence since 1980: systematic analysis of health examination surveys and epidemiological studies with 370 country-years and 2· 7 million participants. *Lancet* 2011; **378**(9785): 31-40.

4. Farzadfar F, Finucane MM, Danaei G, et al. National, regional, and global trends in serum total cholesterol since 1980: systematic analysis of health examination surveys and epidemiological studies with 321 country-years and 3· 0 million participants. *Lancet* 2011; **377**(9765): 578-86.

5. NCD Risk Factor Collaboration (NCD-RisC). Trends in adult body-mass index in 200 countries from 1975 to 2014: a pooled analysis of 1698 population-based measurement studies with 19.2 million participants. *Lancet (London, England)* 2016; **387**(10026): 1377-96.

6. NCD Risk Factor Collaboration (NCD-RisC). Worldwide trends in diabetes since 1980: a pooled analysis of 751 population-based studies with 4.4 million participants. *Lancet (London, England)* 2016; **387**(10027): 1513-30.

7. NCD Risk Factor Collaboration (NCD-RisC). Worldwide trends in blood pressure from 1975 to 2015: a pooled analysis of 1479 population-based measurement studies with 19.1 million participants. *Lancet (London, England)* 2017; **389**(10064): 37-55.

Table S1 – Number of data sources by country, sub-region and risk factor

Country	Number of surveys			Region
	BMI	Blood Pressure	Diabetes	
Canada	17	10	2	North America
United States of America	35	96	32	
Costa Rica	8	7	7	Central Latin America
El Salvador	3	1	1	
Guatemala	7	4	2	
Honduras	4	1	1	
Mexico	20	13	4	
Nicaragua	5	1	1	
Panama	2	1	1	
Antigua y Barbuda	0	0	0	Caribbean (English-speaking)
Bahamas	0	0	0	
Barbados	5	2	4	
Belize	1	1	1	
Bermuda	0	0	0	

Dominica	1	1	1	
Grenada	1	1	0	
Guyana	1	0	0	
Jamaica	5	3	6	
Puerto Rico	4	2	1	
Saint Kitts and Navis	1	1	0	
Saint Lucia	3	2	0	
Saint Vincent and the Grenadines	0	0	0	
Trinidad and Tobago	2	2	0	
Cuba	5	3	3	Caribbean (non- English-speaking)
Dominican Republic	5	2	1	
Haiti	4	0	0	
Suriname	1	0	0	
Bolivia	5	1	1	Andean Latin America
Colombia	12	6	5	
Ecuador	4	2	1	
Peru	20	11	6	
Venezuela	8	7	6	

Argentina	9	6	2	Southern Latin America
Brazil	33	27	9	
Chile	10	6	7	
Paraguay	0	0	1	
Uruguay	4	3	2	

Table S2 - Age-standardised prevalence of obesity (%), prevalence of raised blood pressure (%), and prevalence of diabetes (%) by sub-region in 1980 (95% CrI provided in brackets)

Region	Obesity men	Obesity women	Raised blood pressure men	Raised blood pressure women	Diabetes men	Diabetes women
Andean Latin America	4.92 (3.01-7.69)	12.12 (8.34-16.37)	28.92 (21.40-37.00)	26.70 (19.74-34.93)	4.96 (2.13-10.04)	5.74 (2.55-11.16)
Caribbean (non-English-speaking)	3.94 (2.18-6.31)	12.18 (8.21-16.95)	31.76 (23.35-40.42)	28.97 (21.29-37.89)	4.21 (1.61-8.92)	5.11 (2.03-10.44)
Central Latin America	6.69 (4.04-10.26)	14.48 (9.91-19.76)	28.84 (20.54-37.42)	28.86 (20.94-38.17)	5.97 (2.32-12.14)	6.13 (2.44-12.26)
Caribbean (English-speaking)	4.76 (2.69-7.53)	14.92 (10.41-20.13)	30.73 (22.99-39.05)	26.95 (19.61-35.51)	5.20 (2.09-10.39)	6.40 (2.65-12.75)
North America	12.38 (9.35-16.03)	15.47 (12.17-19.01)	27.58 (22.25-33.20)	19.92 (15.75-24.38)	4.70 (2.25-8.52)	4.29 (2.09-7.94)
Southern Latin America	6.19 (3.96-9.12)	11.46 (7.95-15.71)	37.41 (28.61-46.28)	33.53 (25.68-42.47)	5.60(2.21-11.50)	5.87 (2.38-11.51)

Table S3 - Age-standardised mean body mass index (BMI, kg/m^2), prevalence of obesity (%), mean systolic blood pressure (mmHg), prevalence of raised blood pressure (%), and prevalence of diabetes (%) by sub-region in 2014 (95% CrI provided in brackets)

Region	BMI men	BMI women	Obesity men	Obesity women	Systolic blood pressure men	Systolic blood pressure women	Raised blood pressure men	Raised blood pressure women	Diabetes men	Diabetes women
Andean Latin America	26.11 (25.76-26.46)	26.99 (26.62-27.36)	17.37 (14.36-20.63)	26.38 (23.06-29.81)	125.06 (122.18-127.99)	118.46 (115.41-121.46)	20.21 (15.68-25.1)	15.55 (12.03-19.41)	8.27 (5.13-12.11)	8.62 (5.42-12.55)
Caribbean (non-English-speaking)	25.61 (24.81-26.39)	26.01 (25.44-26.58)	18.56 (14.26-23.26)	30.48 (25.67-35.51)	125.74 (121.25-130.2)	120.92 (116.21-125.7)	23.09 (16.44-30.84)	19.55 (14.02-26.2)	8.01 (4.36-13.03)	9.98 (5.71-16.26)
Central Latin America	27.18 (26.83-27.52)	28.26 (27.91-28.62)	22.22 (18.29-26.27)	31.69 (27.5-36.14)	126.97 (123.71-130.33)	122.25 (118.92-125.6)	22.28 (16.26-29.3)	17.92 (13.01-23.94)	10.41 (5.9-16.42)	11.22 (6.54-17.18)
Caribbean (English-speaking)	26.97 (26.23-27.67)	28.89 (28.18-29.61)	19.21 (15.12-23.47)	34.59 (29.57-39.62)	126.99 (123.08-131.08)	121.1 (116.53-125.57)	24.5 (18.21-31.54)	18.84 (13.83-24.74)	11.11 (6.37-17.28)	13.64 (8.17-21.03)
North America	28.77 (28.37-29.17)	28.7 (28.26-29.15)	34.42 (29.89-39.12)	36.16 (31.58-40.65)	122.98 (120.04-125.96)	116.64 (113.75-119.6)	15.48 (11.12-20.89)	10.71 (7.74-14.49)	7.96 (4.89-12.17)	6.26 (3.8-9.53)
Southern Latin America	26.59 (26.17-27.02)	27.01 (26.52-27.51)	20.05 (16.37-24.08)	26.44 (22.27-30.73)	131.95 (128.82-135.31)	123.33 (120.05-126.75)	27.09 (20.45-34.22)	19.7 (14.78-25.4)	8.32 (4.74-13.36)	8.96 (5.21-14.17)

Table S4 – Prevalence of obesity (%), prevalence of raised blood pressure (%), and prevalence of diabetes (%), for men and women in 1980 and 2014, as well as the ratio of prevalence between 2014 and 1980

	Obesity men			Obesity women			Raised blood pressure men			Raised blood pressure women			Diabetes men			Diabetes women		
	1980	2014	Ratio of prevalence	1980	2014	Ratio of prevalence	1980	2014	Ratio of prevalence	1980	2014	Ratio of prevalence	1980	2014	Ratio of prevalence	1980	2014	Ratio of prevalence
Andean Latin America	4.92	17.37	**3.53**	12.12	26.38	**2.18**	28.92	20.21	**0.70**	26.70	15.55	**0.58**	4.96	8.27	**1.67**	5.74	8.62	**1.50**
Caribbean (non-English-speaking)	3.94	18.56	**4.71**	12.18	30.48	**2.50**	31.76	23.09	**0.73**	28.97	19.55	**0.67**	4.21	8.01	**1.90**	5.11	9.98	**1.95**
Central Latin America	6.69	22.22	**3.32**	14.48	31.69	**2.19**	28.84	22.28	**0.77**	28.85	17.92	**0.62**	5.97	10.41	**1.74**	6.13	11.22	**1.83**
Caribbean (English-speaking)	4.76	19.21	**4.04**	14.92	34.59	**2.32**	30.73	24.50	**0.80**	26.95	18.84	**0.70**	5.20	11.11	**2.14**	6.40	13.64	**2.13**
North America	12.38	34.42	**2.77**	15.47	36.16	**2.34**	27.58	15.48	**0.56**	19.92	10.71	**0.54**	4.70	7.96	**1.69**	4.29	6.26	**1.46**
Southern Latin America	6.19	20.05	**3.24**	11.46	26.44	**2.31**	37.41	27.09	**0.72**	33.53	19.70	**0.59**	5.60	8.32	**1.49**	5.87	8.96	**1.53**

Table S5 - Age-standardised mean body mass index (BMI, kg/m^2), prevalence of obesity (%), mean systolic blood pressure (mmHg), prevalence of raised blood pressure (%), and prevalence of diabetes (%) by country in 2014 (95% CrI provided in brackets)

Country	Sex	Mean BMI	Obesity Prevalence	Mean Systolic Blood Pressure	Prevalence Raised Blood Pressure	Prevalence Diabetes
Argentina	Men	27.81 (26.76-28.89)	27.1 (20.7-34.1)	128.33 (122.74-133.94)	27.9 (19.4-37.7)	9.9 (5.1-16.3)
Argentina	Women	27.57 (26.49-28.68)	29.2 (23.1-35.7)	120.5 (114.97-126.23)	18.2 (12.0-25.2)	9.5 (5.1-15.1)
Antigua and Barbuda	Men	25.65 (23.28-28.08)	11.3 (6.3-17.8)	129.1 (120.59-137.61)	26.3 (16.4-37.6)	9.9 (4.4-17.9)
Antigua and Barbuda	Women	27.7 (24.45-30.85)	25.8 (17.8-34.7)	123.01 (113.58-132.12)	20.2 (12.3-30.0)	13.0 (6.3-22.5)
Bahamas	Men	27.12 (24.79-29.52)	24.1 (16.3-32.4)	128.85 (120.68-137.03)	25.5 (16.7-36.0)	11.2 (5.5-18.7)
Bahamas	Women	28.9 (25.56-32.12)	38.5 (29.8-47.4)	121.12 (112.07-130.18)	17.2 (10.7-25.1)	13.7 (7.2-22.6)
Belize	Men	27.45 (26.13-28.8)	16.0 (10.3-23.2)	125.69 (118.59-132.62)	24.5 (16.1-34.5)	10.0 (4.8-17.1)
Belize	Women	30.51 (29.05-31.92)	31.5 (23.8-39.6)	118.74 (111.11-126.22)	21.1 (13.6-29.9)	15.2 (8.2-24.5)
Bermuda	Men	27.58 (25.19-30.05)	28.7 (20.4-37.6)	128.99 (120.52-137.48)	26.5 (16.6-37.4)	14.2 (7.1-23.9)

Bermuda	Women	29.31 (26.03-32.63)	42.4 (33.4-51.6)	121.31 (112.04-130.33)	17.3 (10.2-26.0)	15.4 (7.9-25.7)
Bolivia	Men	25.21 (23.68-26.7)	14.1 (8.3-21.2)	124.37 (117-131.77)	19.9 (11.9-19.5)	7.0 (2.9-12.4)
Bolivia	Women	27.66 (26.45-28.89)	25.5 (18.7-32.7)	120.07 (111.92-128.34)	16.3 (10.0-24.3)	8.9 (4.1-15.1)
Brazil	Men	26.21 (25.74-26.66)	18.2 (14.2-22.6)	133.17 (129.51-137.2)	27.1 (19.5-35.1)	7.8 (4.1-13.3)
Brazil	Women	26.78 (26.22-27.32)	25.4 (20.9-30.2)	124.3 (120.38-128.35)	20.3 (14.5-26.9)	8.7 (4.6-14.4)
Barbados	Men	26.34 (25.44-27.25)	14.2 (9.3-20.4)	129.76 (124.2-135.34)	27.0 (17.9-37.2)	10.5 (5.1-17.9)
Barbados	Women	29.23 (28.18-30.27)	31.2 (24.1-38.8)	124.73 (119.03-130.17)	21.7 (14.3-30.2)	13.7 (7.2-22.8)
Canada	Men	27.44 (26.99-27.89)	29.2 (24.4-34.2)	118.82 (112.74-124.39)	16.0 (10.0-23.0)	6.2 (3.3-10.3)
Canada	Women	26.7 (26.18-27.21)	29.3 (24.7-34.2)	111.73 (105.79-117.53)	11.1 (7.0-16.0)	4.8 (2.5-7.9)
Chile	Men	27.78 (26.98-28.6)	24.7 (18.7-31.0)	128.32 (123.21-133.52)	25.7 (17.6-34.5)	10.2 (5.5-16.2)
Chile	Women	28.18 (27.23-29.14)	31.2 (25.1-37.7)	119.81 (114.68-124.86)	16.9 (11.4-23.6)	10.8 (6.1-17.0)

Colombia	Men	25.76 (25.08-26.45)	17.3 (12.4-22.6)	126.25 (120.74-132.01)	21.7 (14.5-30.2)	8.3 (4.3-13.8)
Colombia	Women	26.63 (25.87-27.38)	26.7 (21.5-32.1)	119.15 (113.34-125.09)	17.2 (11.2-23.8)	8.7 (4.6-14.1)
Costa Rica	Men	26.77 (26.08-27.48)	20.3 (14.6-26.6)	126.22 (122.29-130.26)	1.3 (14.3-29.3)	8.8 (4.6-14.7)
Costa Rica	Women	27.91 (27.2-28.61)	30.1 (24.0-36.7)	120.27 (116.32-124.27)	16.6 (11.2-22.9)	8.9 (4.8-14.4)
Cuba	Men	25.21 (24.37-26.05)	18.5 (13.0-24.4)	123.52 (118.23-128.85)	21.2 (13.6-30.4)	7.3 (3.5-12.7)
Cuba	Women	26.09 (25.16-27)	30.6 (24.1-37.6)	117.76 (112.43-123.24)	17.2 (11.0-24.9)	9.6 (4.8-16.6)
Dominica	Men	24.91 (23.72-26.11)	19.4 (12.8-26.9)	131.9 (125.27-138.69)	25.9 (17.1-36.1)	8.5 (4.1-14.9)
Dominica	Women	29.02 (27.65-30.42)	35.7 (27.8-43.7)	126.56 (119.62-133.69)	19.7 (12.8-27.7)	13.6 (7.4-22.8)
Dominican Republic	Women	27.36 (26.46-28.24)	33.9 (27.1-41.0)	122.36 (114.49-130.71)	19.5 (12.7-28.3)	11.4 (6.1-19.6)
Dominican Republic	Men	25.55 (24.85-26.28)	20.1 (14.0-26.7)	127.48 (119.87-135.23)	24.1 (15.4-34.2)	8.6 (4.1-15.1)
Ecuador	Men	26.52 (25.74-27.28)	14.5 (9.2-21.1)	124.38 (119.19-129.82)	19.9 (12.1-29.0)	7.5 (3.1-13.2)

Ecuador	Women	27.8 (26.88-28.74)	24.7 (18.1-31.8)	119.15 (113.84-124.58)	16.2 (10.1-23.7)	8.5 (3.7-14.9)
Grenada	Women	28.73 (27.57-29.85)	28.9 (21.1-37.1)	126.61 (120.7-132.56)	21.6 (14.2-30.3)	13.3 (6.7-22.1)
Grenada	Men	25.1 (24.2-26.04)	12.9 (7.8-19.3)	131.85 (125.99-137.7)	26.8 (18.0-37.0)	8.8 (4.0-15.6)
Guatemala	Men	25.67 (24.26-27.02)	14.6 (9.3-20.7)	124.94 (117.99-132.08)	22.0 (14.1-31.5)	8.9 (4.3-15.3)
Guatemala	Women	27.28 (26.55-28.01)	26.2 (20.1-32.7)	119.86 (112.43-127.61)	20.5 (13.1-28.8)	10.4 (5.4-17.2)
Guyana	Men	24.99 (23.95-26.03)	12.1 (7.1-18.3)	127.29 (119.07-135.53)	24.4 (15.3-34.9)	9.1 (4.1-16.3)
Guyana	Women	27.68 (26.39-28.99)	26.8 (19.4-34.7)	123.86 (114.9-132.9)	21.4 (13.5-30.7)	12.6 (6.3-21.5)
Honduras	Men	25.84 (24.12-27.55)	15.1 (9.6-21.7)	126.65 (119.31-134.22)	22.6 (14.5-31.6)	8.5 (4.2-14.4)
Honduras	Women	27.54 (26.68-28.39)	26.5 (20.3-33.3)	120.84 (112.79-128.72)	20.4 (13.2-28.8)	10.0 (5.2-16.6)
Haiti	Men	26.37 (24.04-28.74)	16.8 (10.6-24.4)	127.5 (119.13-135.93)	25.4 (16.2-36.1)	8.6 (4.0-15.5)
Haiti	Women	24.15 (23.15-25.12)	26.4 (20.0-33.3)	124.34 (114.99-133.39)	24.0 (15.6-33.8)	8.8 (4.3-15.9)

Jamaica	Men	25.73 (24.68-26.74)	14.7 (10.0-20.4)	126.39 (120.49-132.52)	24.6 (16.2-34.1)	9.3 (4.5-16.0)
Jamaica	Women	29.54 (28.4-30.72)	33.4 (26.6-40.3)	123.4 (117.23-129.72)	19.4 (12.6-27.4)	14.4 (7.8-23.3)
Saint Kitts and Nevis	Men	28.15 (26.54-29.81)	14.7 (9.1-21.7)	131.28 (124.59-138.19)	28.0 (18.4-38.6)	12.7 (6.0-21.6)
Saint Kitts and Nevis	Women	30.59 (28.67-32.5)	29.9 (21.9-38.4)	127.01 (119.73-134.47)	22.7 (14.8-31.9)	16.3 (8.4-26.6)
Saint Lucia	Men	29.33 (28.46-30.17)	11.5 (6.8-17.3)	131.31 (125.84-136.81)	29.8 (19.9-40.8)	13.7 (6.1-24.1)
Saint Lucia	Women	30.2 (29.14-31.26)	26.6 (19.4-34.4)	125.52 (119.76-131.16)	24.3 (15.9-34.4)	15.1 (7.2-25.9)
Mexico	Men	27.5 (27.13-27.86)	24.0 (19.3-28.8)	127.37 (123.48-131.42)	22.4 (15.4-30.5)	10.9 (5.8-17.7)
Mexico	Women	28.44 (28.01-28.87)	32.9 (28.0-38.1)	122.96 (119.02-126.92)	17.6 (12.0-24.4)	11.5 (6.4-18.3)
Nicaragua	Men	26.53 (24.84-28.29)	17.5 (11.4-24.4)	126.81 (119.28-134.19)	21.7 (13.9-30.8)	9.2 (4.6-15.7)
Nicaragua	Women	27.94 (26.75-29.13)	28.9 (22.5-35.6)	122.47 (114.69-130.35)	20.1 (13.0-28.4)	11.0 (5.8-17.8)
Panama	Men	26.19 (25.02-27.37)	17.4 (11.9-23.8)	127.83 (121.73-133.75)	22.4 (15.2-31.2)	8.9 (4.6-14.7)

Panama	Women	27.67 (26.39-28.93)	27.6 (21.1-34.6)	121.72 (115.33-127.85)	17.5 (11.5-24.6)	9.8 (5.3-15.6)
Peru	Men	26.11 (25.73-26.48)	14.9 (10.8-19.2)	123.94 (121.27-126.62)	16.4 (11.4-22.1)	7.2 (3.2-12.6)
Peru	Women	27.07 (26.71-27.44)	24.2 (20.1-28.7)	115.87 (113.27-118.59)	11.6 (8.0-15.7)	8.1 (3.8-13.7)
Puerto Rico	Men	28.33 (27.05-29.6)	27.9 (20.6-35.8)	125.57 (118.36-132.68)	22.6 (14.3-32.3)	13.0 (6.6-21.6)
Puerto Rico	Women	28.53 (27.35-29.74)	40.8 (33.4-48.4)	117.89 (109.9-125.92)	15.9 (9.8-23.8)	13.4 (7.1-22.4)
Paraguay	Men	26.48 (23.97-29)	16.7 (10.4-24.5)	129.35 (120.99-137.81)	28.1 (18.6-38.8)	8.0 (3.8-14.0)
Paraguay	Women	26.88 (23.47-30.2)	23.3 (15.7-31.7)	122.57 (113.37-131.28)	21.5 (13.8-30.7)	8.3 (4.2-14.1)
El Salvador	Men	26.83 (25.13-28.65)	18.4 (12.0-25.6)	124.22 (116.8-131.59)	20.6 (12.9-29.1)	9.3 (4.8-15.6)
El Salvador	Women	28.28 (27.14-29.41)	28.8 (22.2-35.9)	118.66 (110.53-126.59)	17.4 (10.9-25.2)	10.7 (5.7-17.1)
Suriname	Men	25.38 (24.09-26.64)	18.6 (12.4-25.7)	127.72 (119.54-136.05)	24.8 (16.0-35.5)	10.9 (5.5-18.3)
Suriname	Women	27.76 (26.1-29.34)	34.0 (26.5-41.7)	122.89 (113.66-131.76)	20.2 (13.0-28.7)	13.5 (7.3-22.6)

Trinidad and Tobago	Men	26.6 (25-28.13)	10.3 (5.7-17.0)	129.08 (121.42-136.64)	27.4 (17.4-39.1)	10.4 (4.5-19.1)
Trinidad and Tobago	Women	28.62 (26.69-30.44)	25.6 (17.7-34.3)	122.9 (114.67-130.95)	23.7 (15.0-33.8)	13.0 (6.0-22.7)
Uruguay	Men	27.28 (26.29-28.24)	24.7 (18-0.32)	127.04 (121.91-131.97)	25.3 (17.2-34.0)	9.1 (4.8-14.9)
Uruguay	Women	27.38 (26.26-28.5)	30.9 (23.9-38.3)	120.47 (115.11-125.83)	17.2 (11.4-24.1)	9.8 (5.3-15.9)
United States of America	Men	28.93 (28.49-29.36)	35.0 (30.1-40.1)	123.46 (120.36-126.68)	15.4 (10.8-21.3)	8.2 (4.9-12.7)
United States of America	Women	28.92 (28.45-29.42)	36.9 (32.1-41.8)	117.2 (114.01-120.45)	10.7 (7.5-14.7)	6.4 (3.8-9.9)
Saint Vincent and the Grenadines	Men	26.24 (23.94-28.62)	15.9 (9.8-23.2)	128.76 (120.45-136.81)	26.0 (16.9-36.0)	9.3 (4.5-16.3)
Saint Vincent and the Grenadines	Women	28.19 (24.96-31.41)	30.9 (22.7-39.7)	122.88 (114-131.7)	20.9 (13.4-30.1)	11.8 (6.1-19.9)
Venezuela	Women	26.89 (26.19-27.59)	29.0 (23.2-35.3)	118.86 (112.91-124.5)	16.1 (10.5-22.7)	9.0 (4.8-14.4)
Venezuela	Men	26.69 (26.06-27.3)	22.3 (16.6-28.3)	124.86 (119.11-130.32)	21.9 (14.6-30.3)	9.9 (5.3-15.7)

Figure S1 – Mean body mass index and systolic blood pressure by sub-regions in men

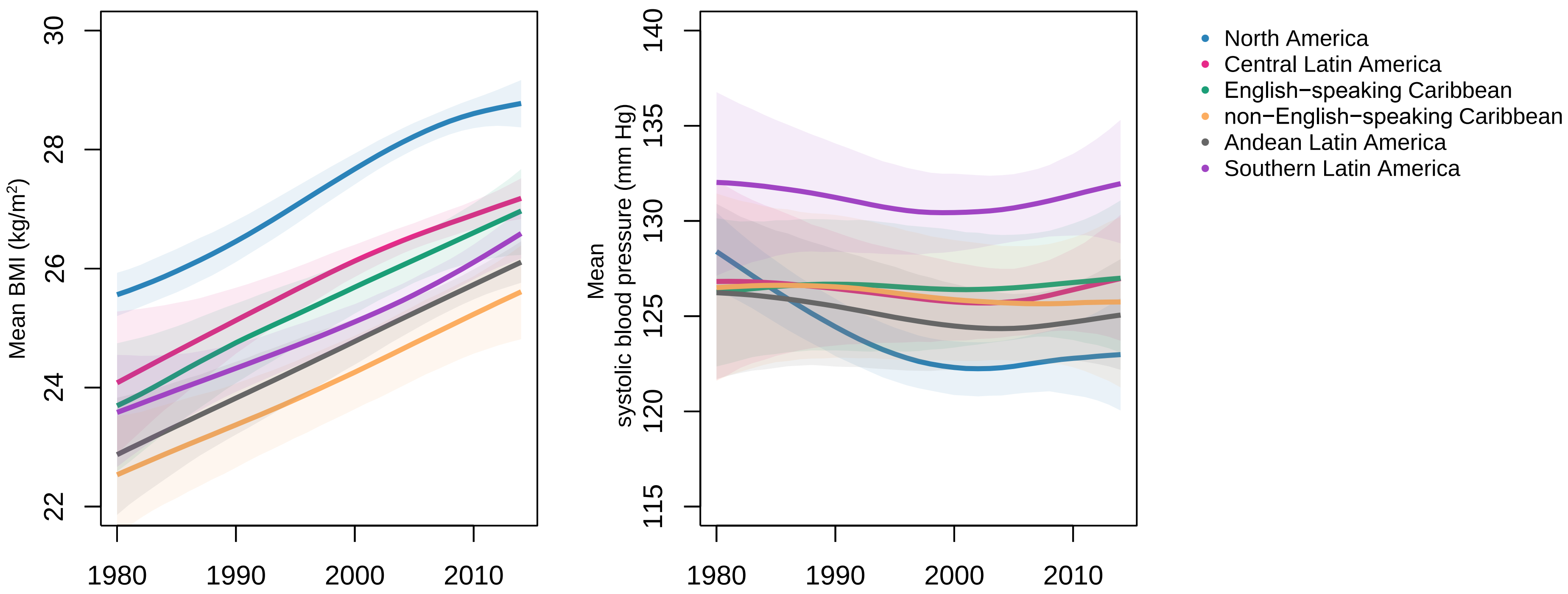

Mean BMI (kg/m^2)
30
28
26
24
22
1980
1990
2000
2010
Mean
systolic blood pressure (mm Hg)
140
135
130
125
120
115
1980
1990
2000
2010
North America
Central Latin America
English−speaking Caribbean
non−English−speaking Caribbean
Andean Latin America
Southern Latin America

Figure S2 – Mean body mass index and systolic blood pressure by sub-regions in women

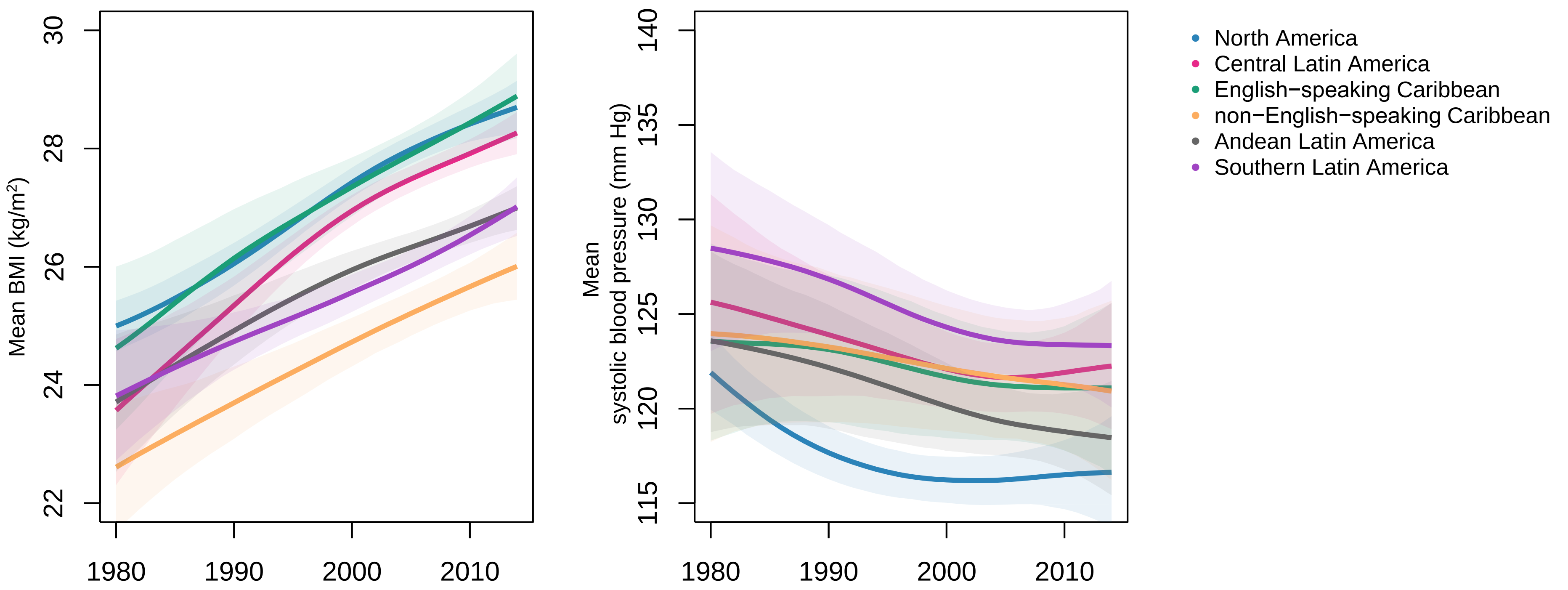

Mean BMI (kg/m2)
22
24
26
28
30
1980
1990
2000
2010
Mean
systolic blood pressure (mm Hg)
115
120
125
130
135
140
1980
1990
2000
2010
North America
Central Latin America
English−speaking Caribbean
non−English−speaking Caribbean
Andean Latin America
Southern Latin America

Figure S3 – Heatmap of age-standardised prevalence of obesity, raised blood pressure (RBP), and diabetes (DM) by country in (A) men and (B) women in 1980. Countries ranked by prevalence of obesity

A

	Obesity (%)	Raised blood pressure (%)	Diabetes (%)
Bermuda	13.4	47.6	10.6
United States of America	12.6	27	4.7
Argentina	11	43	7.4
Canada	10.7	33.3	5
Chile	9.9	37.9	7.2
Uruguay	9.6	43.2	7.5
Venezuela	7.9	34.9	7
Mexico	7.7	29.9	6.5
Bahamas	7.5	36.9	6.3
Puerto Rico	6.9	33.5	6.8
Suriname	5.1	33.1	6.1
Belize	4.9	33.2	5.4
Cuba	4.8	36.2	5
Nicaragua	4.8	28.6	5.1
Colombia	4.5	29.2	4.4
Peru	4.3	26.7	4.9
Brazil	4.3	35.6	4.8
Panama	4.2	27.8	4.5
Barbados	4.1	33	5
El Salvador	4	25.4	4.9
Costa Rica	3.8	26.3	4.2
Dominican Republic	3.7	28.6	3.5
Dominica	3.7	30.7	4
Saint Kitts and Nevis	3.7	29.9	5.4
Paraguay	3.6	30	3.4
Ecuador	3.3	23.6	3.7
Guatemala	3.3	24.8	4.4
Jamaica	3.2	30.3	3.9
Antigua and Barbuda	3	27.5	3.9
Saint Vincent and the Grenadines	3	28	4.1
Grenada	2.9	28	3.4
Bolivia	2.9	25.8	4.1
Honduras	2.9	24.1	3.7
Guyana	2.7	26.8	3.6
Saint Lucia	2.7	26.2	3.4
Trinidad and Tobago	2.1	23.6	3.2
Haiti	2	24.7	2.8

B

	Obesity (%)	Raised blood pressure (%)	Diabetes (%)
Bermuda	31.2	40.4	11.2
Bahamas	21.1	31.5	8
Puerto Rico	18.6	30	7.3
Uruguay	16.8	35.9	7.3
Chile	16.8	32.9	7.8
Suriname	16.4	30.5	7.8
Mexico	15.9	30.2	6.5
Venezuela	15.8	32.5	6.9
United States of America	15.8	19.5	4.3
Belize	15.5	29.3	7.6
Argentina	15.4	34.5	6.1
Cuba	15	31.7	6.2
Barbados	14.2	27.3	7.2
Jamaica	13.9	26.1	5.7
Dominica	13.4	27.2	6.3
Canada	12.7	24.1	4
Dominican Republic	12.4	27.6	4.6
Nicaragua	12.3	27.7	5.8
Saint Kitts and Nevis	12.3	24.7	7.2
Colombia	11.9	27	5.6
Peru	11.4	24.2	5.9
Panama	10.8	24.7	5.1
Grenada	10.8	24.2	5.1
Antigua and Barbuda	10.7	22.2	5.6
Saint Vincent and the Grenadines	10.6	24.3	5.3
El Salvador	10.4	24.1	5.5
Guyana	10	23.5	5
Saint Lucia	9.8	22.5	4.8
Costa Rica	9.7	23.7	4.4
Ecuador	9.5	22.4	4.5
Brazil	9.5	33.4	5.6
Guatemala	9.5	24.7	5
Bolivia	9.1	23.9	4.9
Honduras	8.5	23.6	3.9
Trinidad and Tobago	7.5	19.9	4.9
Paraguay	7.1	25.9	3.6
Haiti	6	24.5	3

Figure S4 – Age standardised prevalence of obesity vs. age standardised prevalence of raised blood pressure and age standardised prevalence of diabetes by sex, (A) 1980 and (B) 2014

A

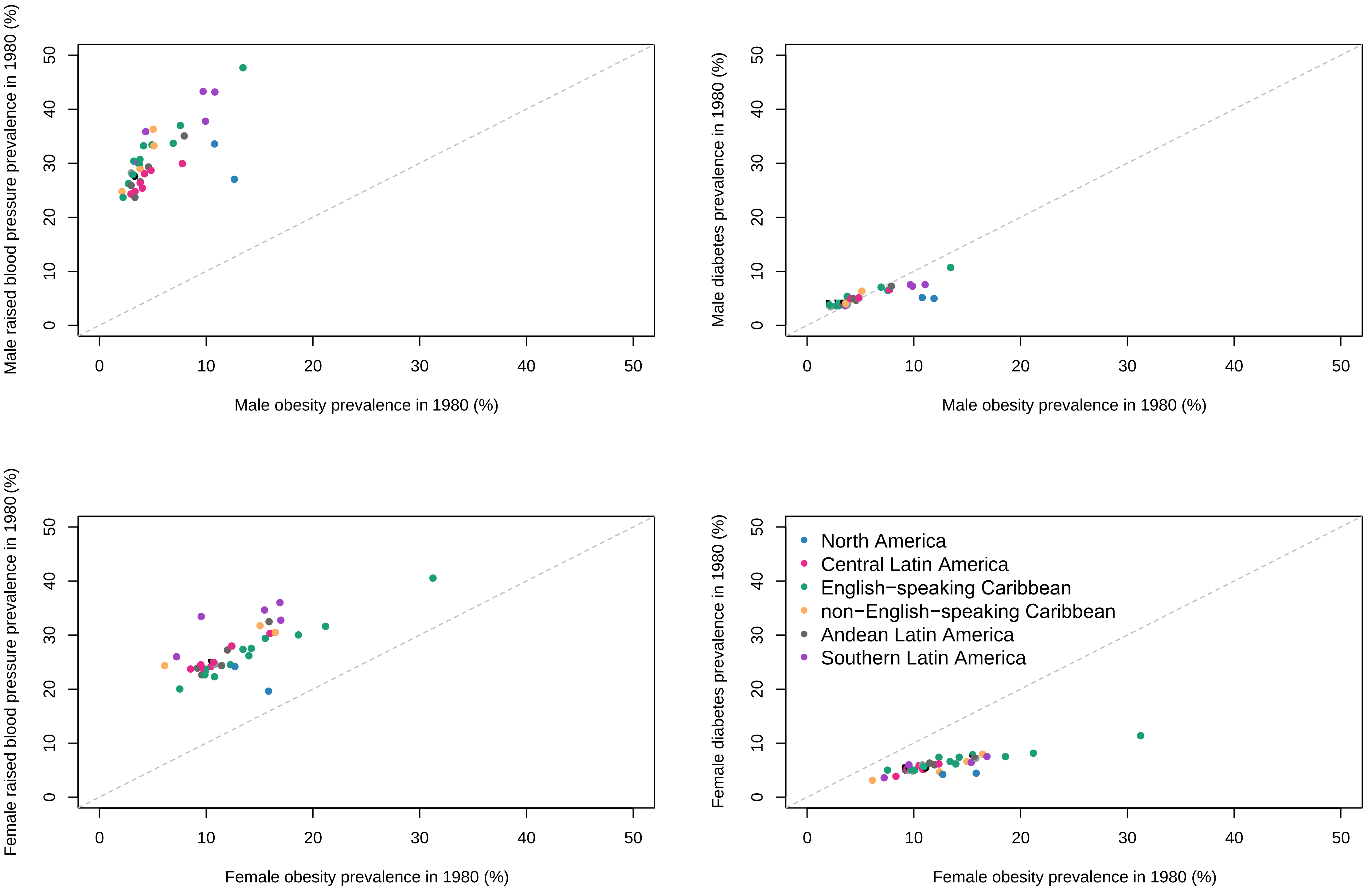
Male raised blood pressure prevalence in 1980 (%)
Male obesity prevalence in 1980 (%)
Male diabetes prevalence in 1980 (%)
Male obesity prevalence in 1980 (%)
Female raised blood pressure prevalence in 1980 (%)
Female obesity prevalence in 1980 (%)
Female diabetes prevalence in 1980 (%)
Female obesity prevalence in 1980 (%)
North America
Central Latin America
English−speaking Caribbean
non−English−speaking Caribbean
Andean Latin America
Southern Latin America
0
10
20
30
40
50

B

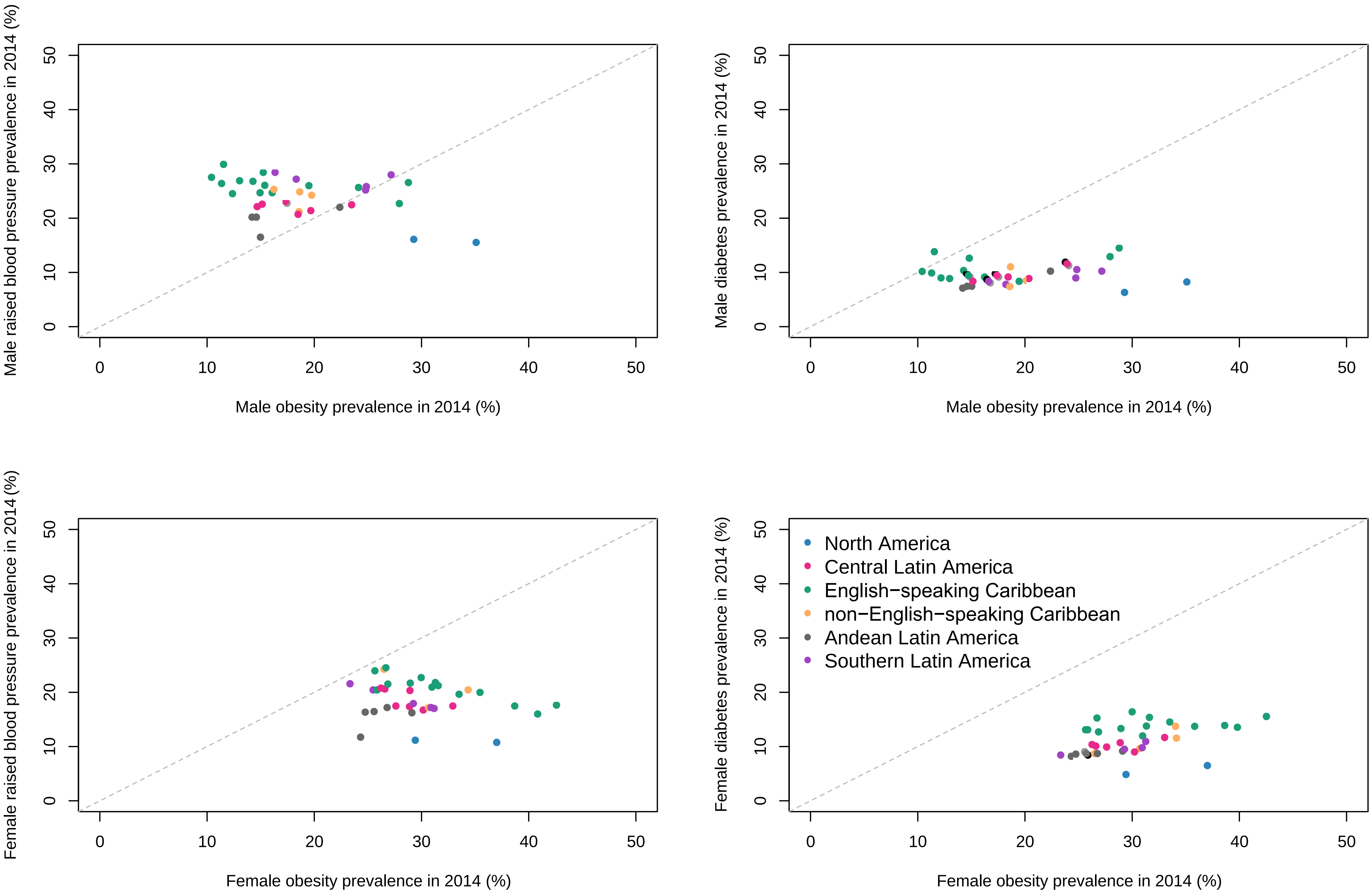
Male raised blood pressure prevalence in 2014 (%)
Male obesity prevalence in 2014 (%)
Male diabetes prevalence in 2014 (%)
Male obesity prevalence in 2014 (%)
Female raised blood pressure prevalence in 2014 (%)
Female obesity prevalence in 2014 (%)
Female diabetes prevalence in 2014 (%)
Female obesity prevalence in 2014 (%)
North America
Central Latin America
English−speaking Caribbean
non−English−speaking Caribbean
Andean Latin America
Southern Latin America
0
10
20
30
40
50
